# Supplementary material for: Comparing Prognosis for BRCA1, BRCA2, and Non-BRCA Breast Cancer
Source: Cancers (Basel). 2023 Dec 3;15(23):5699. doi: 10.3390/cancers15235699 (PMC10705326; doi:10.3390/cancers15235699)
Supplement: Supplementary file 1 [file cancers-15-05699-s001.zip › cancers-2730210-supplementary.pdf]

Table S1. Cox Regression

Case Processing Summary

		N	Percent
Cases available in analysis	Event[a]	45	6.6%
	Censored	0	0.0%
	Total	45	6.6%
Cases dropped	Cases with missing values	639	93.4%
	Cases with negative time	0	0.0%
	Censored cases before the earliest event in a stratum	0	0.0%
	Total	639	93.4%
Total		684	100.0%

a. Dependent Variable: Invasive Disease-Free Survival (months)

Categorical Variable Codings[a,c,d,e]

		Frequency	(1)	(2)	(3)
Subtype[b]	1=Luminal A	27	0	0	0
	2=Luminal B	6	1	0	0
	3=HER2 enriched	2	0	1	0
	4=Triple Negative	10	0	0	1
Mastectomy[b]	0=No	28	0		
	1=Yes	17	1		
Oophorectomy[b]	0=No	17	0		
	1=Yes	28	1		
mastectomy_and_oophorectomy[b]	0=No	31	0		
	1=Yes	14	1		

a. Category variable: Subtype

b. Indicator Parameter Coding

c. Category variable: Mastectomy

d. Category variable: Oophorectomy

e. Category variable: mastectomy_and_oophorectomy

Block 1: Method = Forward Stepwise (Conditional LR)

Variables in the Equation

		B	SE	Wald	df	Sig.	Exp(B)	95.0% CI for Exp(B)	
								Lower	Upper
Step 1	Oophorectomy	-.899	.327	7.552	1	.006	.407	.215	.773

Variables not in the Equation[a]

		Score	df	Sig.
Step 1	Mastectomy	.127	1	.722
	mastectomy_and_oophorectomy	2.646	1	.104
	Subtype	.098	3	.992
	Subtype(1)	.009	1	.926
	Subtype(2)	.031	1	.860
	Subtype(3)	.059	1	.809
	Age_diagnosis	.480	1	.488

a. Residual Chi Square = 6.011 with 6 df Sig. = .422

Cox Regression

Case Processing Summary

		N	Percent
Cases available in analysis	Event[a]	24	3.5%
	Censored	213	31.1%
	Total	237	34.6%
Cases dropped	Cases with missing values	447	65.4%
	Cases with negative time	0	0.0%
	Censored cases before the earliest event in a stratum	0	0.0%
	Total	447	65.4%
Total		684	100.0%

a. Dependent Variable: Overall Survival (meses)

Categorical Variable Codings[a,c,d,e]

		Frequency	(1)	(2)	(3)
Subtype[b]	1=Luminal A	139	0	0	0
	2=Luminal B	18	1	0	0
	3=HER2 enriched	4	0	1	0
	4=Triple Negative	76	0	0	1
Mastectomy[b]	0=No	78	0		
	1=Yes	159	1		
Oophorectomy[b]	0=No	55	0		
	1=Yes	182	1		
mastectomy_and_oophorectomy[b]	0=No	96	0		
	1=Yes	141	1		

a. Category variable: Subtype

b. Indicator Parameter Coding

c. Category variable: Mastectomy

d. Category variable: Oophorectomy

e. Category variable: mastectomy_and_oophorectomy

Block 1: Method = Forward Stepwise (Conditional LR)

Variables in the Equation

		B	SE	Wald	df	Sig.	Exp(B)	95.0% CI for Exp(B)	
								Lower	Upper
Step 1	Mastectomy	-1.624	.449	13.067	1	.000	.197	.082	.475

Variables not in the Equation[a]

		Score	df	Sig.
Step 1	Oophorectomy	3.658	1	.056
	mastectomy_and_oophorectomy	.093	1	.760
	Subtype	5.099	3	.165
	Subtype(1)	3.822	1	.051
	Subtype(2)	.506	1	.477
	Subtype(3)	.174	1	.677
	Age_diagnosis	.155	1	.693

a. Residual Chi Square = 10.327 with 6 df Sig. = .112
